# CK1α/RUNX2 Axis in the Bone Marrow Microenvironment: A Novel Therapeutic Target in Multiple Myeloma

**DOI:** 10.3390/cancers14174173

**Published:** 2022-08-29

**Authors:** Anna Fregnani, Lara Saggin, Ketty Gianesin, Laura Quotti Tubi, Marco Carraro, Gregorio Barilà, Greta Scapinello, Giorgia Bonetto, Maria Pesavento, Tamara Berno, Antonio Branca, Carmela Gurrieri, Renato Zambello, Gianpietro Semenzato, Livio Trentin, Sabrina Manni, Francesco Piazza

**Affiliations:** 1 Hematology and Clinical Immunology Branch, Department of Medicine, University of Padova, 35128 Padova, Italy; 2 Laboratory of Myeloma and Lymphoma Pathobiology, Veneto Institute of Molecular Medicine (VIMM), 35129 Padova, Italy

**Keywords:** multiple myeloma, bone marrow microenvironment, CK1α, RUNX2, target therapy

## Abstract

**Simple Summary:**

Multiple myeloma (MM) is an incurable disease for which novel therapeutic approaches targeting the malignant cells and the associated bone disease are urgently needed. CK1α is a protein kinase that plays a crucial role in the signaling network that sustains plasma cell (PC) survival and bone disease. This protein regulates Wnt/β-catenin signaling, which is fundamental for both MM cell survival and mesenchymal stromal cell (MSC) osteogenic differentiation. In this study, we investigated its involvement in MM–MSC cross-talk. We found that, by lowering CK1α expression levels in co-cultures of MM and MSC cells, expression of RUNX2—the master regulator of osteogenic differentiation—was regulated differently in the two cell types. Our data suggest the possibility of using a specific CK1α inhibitor as part of a novel therapeutic approach to selectively kill malignant PCs and overcome the blocking of osteogenic differentiation induced by MM cells in MSCs.

**Abstract:**

Multiple myeloma (MM) is a malignant plasma cell (PC) neoplasm, which also displays pathological bone involvement. Clonal expansion of MM cells in the bone marrow causes a perturbation of bone homeostasis that culminates in MM-associated bone disease (MMABD). We previously demonstrated that the S/T kinase CK1α sustains MM cell survival through the activation of AKT and β-catenin signaling. CK1α is a negative regulator of the Wnt/β-catenin cascade, the activation of which promotes osteogenesis by directly stimulating the expression of *RUNX2*, the master gene regulator of osteoblastogenesis. In this study, we investigated the role of CK1α in the osteoblastogenic potential of mesenchymal stromal cells (MSCs) and its involvement in MM–MSC cross-talk. We found that CK1α silencing in in vitro co-cultures of MMs and MSCs modulated RUNX2 expression differently in PCs and in MSCs, mainly through the regulation of Wnt/β-catenin signaling. Our findings suggest that the CK1α/RUNX2 axis could be a potential therapeutic target for constraining malignant PC expansion and supporting the osteoblastic transcriptional program of MSCs, with potential for ameliorating MMABD. Moreover, considering that Lenalidomide treatment leads to MM cell death through Ikaros, Aiolos and CK1α proteasomal degradation, we examined its effects on the osteoblastogenic potential of MSC compartments.

## 1. Introduction

Multiple myeloma (MM) is an incurable hematological malignancy of terminally differentiated plasma cells (PCs) which accounts for about 1% of all cancers, being the second most common hematological malignant tumor after non-Hodgkin lymphoma (NHL) [1]. The disease is characterized by the uncontrolled growth of clonal PCs in the bone marrow (BM), which leads to the over-secretion of non-functional monoclonal immunoglobulins (Igs) [2]. In the BM tumor microenvironment (TME), the secretion of soluble factors and cell–cell contacts between PCs and BM stromal cells constitute the main features of the disease, i.e., the deregulation of bone homeostasis and the establishment of a permissive growth-promoting *niche* for malignant PCs [3]. Together with the accumulation of monoclonal Igs or parts of them, the growth of malignant PCs in the BM is responsible for the clinical features of the disease, summarized in the acronym “CRAB”: calcium (elevated), renal injury, anemia and bone disease [4]. In particular, the perturbation of bone homeostasis gives rise to MM-associated bone disease (MMABD), which is characterized by skeletal-related events (SREs) in 80–90% of patients during their tumor course and negatively impacts quality of life and survival [5]. Physiologically, bone homeostasis is tightly regulated by the action of different coordinated signaling pathways that balance bone formation and resorption [6]. In the pathological MM TME, a simultaneous increase in osteoclast proliferation/activity and inhibition of osteoblasts drives MMABD development [7,8,9,10,11]. The transcription factor *RUNX2* is the master gene regulator of skeletogenesis, as it tunes the MSC commitment towards the osteoblastic lineage. RUNX2 activation in human MSCs stimulates the expression of the earlier osteoblastic markers Osterix (OSX), Alkaline phosphatase (ALP), Collagen type Iα1 (COL1α1) and, later, Osteopontin (OPN) and Osteocalcin (OCN) [12]. Indeed, the *COL1A1*, *SPP1* and *BGLAP* genes, respectively coding for COL1α1, OPN and OCN, present in their promoters a specific sequence for the RUNX2 DNA-binding site [13,14]. Interestingly, the inhibitory effects of MM cells on osteoblast differentiation appear to be mediated, in part, by the capability of MM cells to counteract RUNX2 activity in MSCs and osteoprogenitor cells [15,16]. Moreover, evidence has confirmed higher levels of *RUNX2* m-RNA and proteins in primary MM cells than in PCs isolated from healthy/MGUS donors [17]. Furthermore, RUNX2 activity in MM cells sustains angiogenesis, cell survival and tumor progression [18]. Indeed, RUNX2 knockdown in MM cells significantly reduces different patterns of cytokines, chemokines and growth factors involved in tumor progression and bone metastasis, thus counteracting MMABD progression [17].

Wnt/β-catenin signaling is one of the major pathways that controls RUNX2 expression, thus promoting osteogenesis. A functional T cell factor (TCF) regulatory element responsive to canonical Wnt signaling resides in the promoter of the *RUNX2* gene, suggesting a relationship of direct regulation between the canonical Wnt pathway and osteogenesis [19]. Importantly, Wnt/β-catenin signaling is necessary in the early phases of bone formation [20,21]. Oppositely, in the later phases, the Wnt pathway needs to be downregulated to allow the final maturation of osteoblasts, favoring bone mineralization [20,21]. 

Protein kinase CK1α, the smallest (37 kDa) isoform of the CK1 family, is known to negatively regulate Wnt/β-catenin signaling through the phosphorylation of β-catenin on Ser 45, priming it for subsequent proteasomal degradation [22]. Moreover, PC survival and proliferation are “addicted” to CK1α function [23]. Indeed, this pleiotropic kinase regulates different molecular pathways involved in the control of cell fate [24], such as PI3K/AKT [23] and NF-κB signaling cascades [25], as well as apoptotic signaling pathways [26] and cell cycle progression [27]. We previously demonstrated that CK1α is overexpressed in mantle cell lymphoma [28] and in MM [23] and that its inactivation causes MM PC apoptosis and cell cycle arrest through the regulation of AKT, β-catenin and p53 protein levels [23]. Moreover, CK1α inhibition can cause MM cell death, also, through the downregulation of pro-survival autophagy [29]. 

Lenalidomide (Lena), an immune-modulating drug (IMiD) currently used in MM therapy, acts by directing Cereblon (CRBN) E3 ubiquitin ligase towards specific substrates. In MM cells, Lena induces the ubiquitination and subsequent proteasomal degradation of Ikaros, Aiolos [30] and CK1α [23], leading to MM cell death [23]. Moreover, CK1α targeting boosts Lena-induced cytotoxicity in MM cells [23]. Although Lena’s mechanisms of action in MM PCs have been intensively studied, its effects on the stromal compartment in the BM *niche* are controversial. The impact of IMiDs on MSC differentiation and on bone remodeling is still debated [31,32,33,34,35,36]. Therefore, it is necessary to investigate Lena’s effects on osteoblastic-related gene expression.

Even if the pro-survival/oncogenic role of this kinase is clear for MM PCs [23,37], nothing is known about the role of this kinase with respect to the osteoblastic potential of stromal cells and therefore of the impact of targeting it to mitigate MMABD. The known role of CK1α on Wnt/β-catenin signaling regulation in different cells type [22,38] and the fundamental role of β-catenin on osteoblastogenesis through the regulation of RUNX2 [19,39,40] prompted us to explore whether CK1α could modulate the osteoblastogenic potential of stromal cells through the regulation of the Wnt/β/catenin/RUNX2 axis in different CK1α loss-of-function cell models of the MM BM microenvironment, including an analysis of MM plasma cells and stromal cells. In this context, CK1α could represent a new therapeutic target for the dual causation of MM cell death and mitigation of MMABD.

## 2. Materials and Methods

### 2.1. Patients and Cell Cultures

INA-6 or H929 MM cells and HS-5 cell lines were obtained and cultured as in [23]. MSC-hTERT cells were described in [41]. Primary MGUS and MM BMSCs were isolated and cultured as previously described [42]. Patient samples were processed and used after written informed consent was obtained, according to the Declaration of Helsinki. 

### 2.2. RNA Interference

The *CSNK1A1*-directed shRNA IPTG-inducible INA-6 6044 MM cells and the MSC-hTERT 6044 cellular clones were described in [23,41], respectively, and generated by *CSNK1A1*-specific shRNA (pLKO_IPTG_3XLacO, with the sequence code TRCN0000006044) lentiviral transduction. HS-5 cells (30,000 cells/well in a 48-well plate) were transduced with *CSNK1A1*-specific shRNA (pLKO_IPTG_3XLacO, sequence code TRCN0000006044) lentiviral particles at 37 °C, 5% CO_2_, overnight, in DMEM growing medium with 8 µg/mL polybrene (Sigma-Aldrich, Milan, Italy), with a multiplicity of infection of 3. After 24 h, the medium was replaced, and cells were grown in a complete DMEM medium without polybrene. Puromycin selection (0.5 µg/mL) was started two days after transduction to obtain the HS-5 6044 cellular clone. An antibiotic kill curve assay was performed to obtain a titration curve for puromycin resistance. To induce *CSNK1A1* gene silencing, cells were incubated with IPTG 500 µM, refreshed every two days for different time periods.

### 2.3. Co-Culture Models

Co-cultures of MM cells and stromal cells were obtained by culturing the stromal cell line MSC-hTERT and the IL-6-dependent MM INA-6 cell line as described in [42]. The co-culture phase was preceded by a pre-induction phase, in which IPTG 500 µM was added to INA-6 WT/INA-6 6044 or MSC-hTERT 6044 cells, for seven days. To obtain the co-culture, 2.0 × 10^6^ MM cells were seeded on a feeder layer of stromal cells, and the co-culture was stabilized for an additional three days before harvesting. The IPTG treatment was maintained for the same duration, for a total of ten days. At the end of the co-culture period, cell sorting by FACSARIA IIIu (Becton-Dickinson, Franklin Lakes, NJ, USA) was used to obtain MSC and MM pure populations. In some co-culture experiments, cell–cell contacts were disrupted using a transwell system (Greiner Bio-one, Milan, Italy), where 0.4 µm cell culture porous inserts were placed between the PCs and the stromal cells. 

### 2.4. Cell Sorting

MSC-hTERT cells have been genetically modified to express Green Fluorescence Protein (GFP) [43]. Cell sorting was performed using a FACSARIA IIIu cytometer and FACS Diva software (Becton-Dickinson, Franklin Lakes, NJ, USA) to discriminate GFP-positive MSC-hTERT cells from GFP-negative INA-6 cells in the different MSC-hTERT–INA-6 co-culture models. 

### 2.5. Wnt-3A Stimulation

For 72 h, 3 × 10^5^ HS-5 6044 cells and MSC-hTERT 6044 cells were respectively plated and cultured to allow cell adhesion and cell growth. Cell medium (RPMI 1640 with hydrocortisone 1 μM for MSC-hTERT cells and DMEM for HS-5 cells, both with 10% FBS and antibiotics (penicillin 100U/mL and streptomycin 100 μg/mL)) was changed with starvation medium (RPMI 1640/DMEM with 5% FBS and antibiotics added) for 24h. After the starvation phase, both stromal cell lines were treated with Recombinant Human WNT-3A Protein (R&D System, Minneapolis, MN, USA) at final concentrations of 200 ng/mL for 8 h (MSC-hTERT cells) and for 4 h (HS-5 cells), as described in [44]. 

### 2.6. Chemicals

Lenalidomide was purchased from Selleck chemicals (Houston, TX, USA); Isopropyl-b-D-1-thiogalattopiranoside (IPTG) and Doxorubicin were purchased from Sigma-Aldrich (Sigma-Aldrich, Milan, Italy). 

### 2.7. Evaluation of Apoptosis and Cell Viability

Apoptosis was assessed by staining with Annexin V/Propidium Iodide (PI) (purchased from IMMUNOSTEP, Salamanca, Spain) and FACS analysis, as in [28]. Viability was assessed by Trypan Blue exclusion dye analysis assay. 

### 2.8. Cell Cycle Analysis

The cell cycle analysis was performed as described in [28] and analyzed using a FACS-CANTO II Cell Cytometer and FACS Diva software (Becton-Dickinson, Italy). 

### 2.9. Western Blot (WB)

WB analysis was performed as described in [45]. The antibodies used were: CK1α, PARP, β-Catenin (Cell signaling Technology, Danvers, MA, USA); GAPDH (Millipore, Milan, Italy); RUNX2, p21 (Santa Cruz Biotechnology, Dallas, TX, USA); and p53 (Thermo Fisher, Waltham, MA, USA). Images were taken with the Image Quant LAS 500 chemiluminescence detection system (GE Healthcare, Chicago, IL, USA), and densitometric analysis of the bands was performed using ImageQuantTL software. The original WB is presented in the Supplementary Materials (Figure S2).

### 2.10. Quantitative Real-Time PCR

Quantitative real-time PCR was performed as in [46], using the QuantStudio 5 detection system (Applied Biosystem, Foster City, CA, USA) with QuantStudioTM Design and Analysis Software v.1.4.3. The primers used were the following: 

*RUNX2* Forward 5′-3′ TAAGAAGAGCCAGGCAGGTG and Reverse 5′-3′ TAGTGCATTCGTGGGTTGG; *ALP* Forward 5′-3′ GACCCTTGACCCCCACAAT and Reverse 5′-3′ GCTCGTACTGCATGTCCCCT; *SPP1* Forward 5′-3′ CTCAGGCCAGTTGCAGCC and Reverse 5′-3′ CAAAAGCAAATCACTGCAATTCTC; *BGLAP* Forward 5′-3′ GAAGCCCAGCGGTGCA and Reverse 5′-3′ CACTACCTCGCTGCCCTC; *CSNK1A1* Forward 5′-3′ GGCACTGCCCGATATGCTA and Reverse 5′-3′ CTCGGCGACTCTGCTCAATAC; *AXIN2* Forward 5′-3′ GTGTGAGGTCCACGGAAACT and Reverse 5′-3′ TGGCTGGTGCAAAGACATAG; *GAPDH* Forward 5′-3′ AATGGAAATCCCATCACCATCT and Reverse 5′-3′ CGCCCCACTTGATTTTGG. 

### 2.11. Alkaline Phosphatase Assay

ALP activity was analyzed in medium supernatants collected after Lena treatment of primary MSCs isolated from MM/MGUS patient samples using the colorimetric alkaline phosphatase assay kit (ab83369, Abcam, Cambridge, UK), following the manufacturer’s instructions. 

### 2.12. Statistical Analysis

Data normality was analyzed using the Shapiro–Wilk test. Data were analyzed for statistical significance with the two-tailed unpaired Student’s *t*-test using GraphPad Prism 8.0.1 software. Statistical significance was considered with *p*-values below 0.05.

## 3. Results

### 3.1. CK1α Silencing in Stromal Cells Leads to the Upregulation of Β-Catenin and Osteogenic Markers

**Figure 1:**
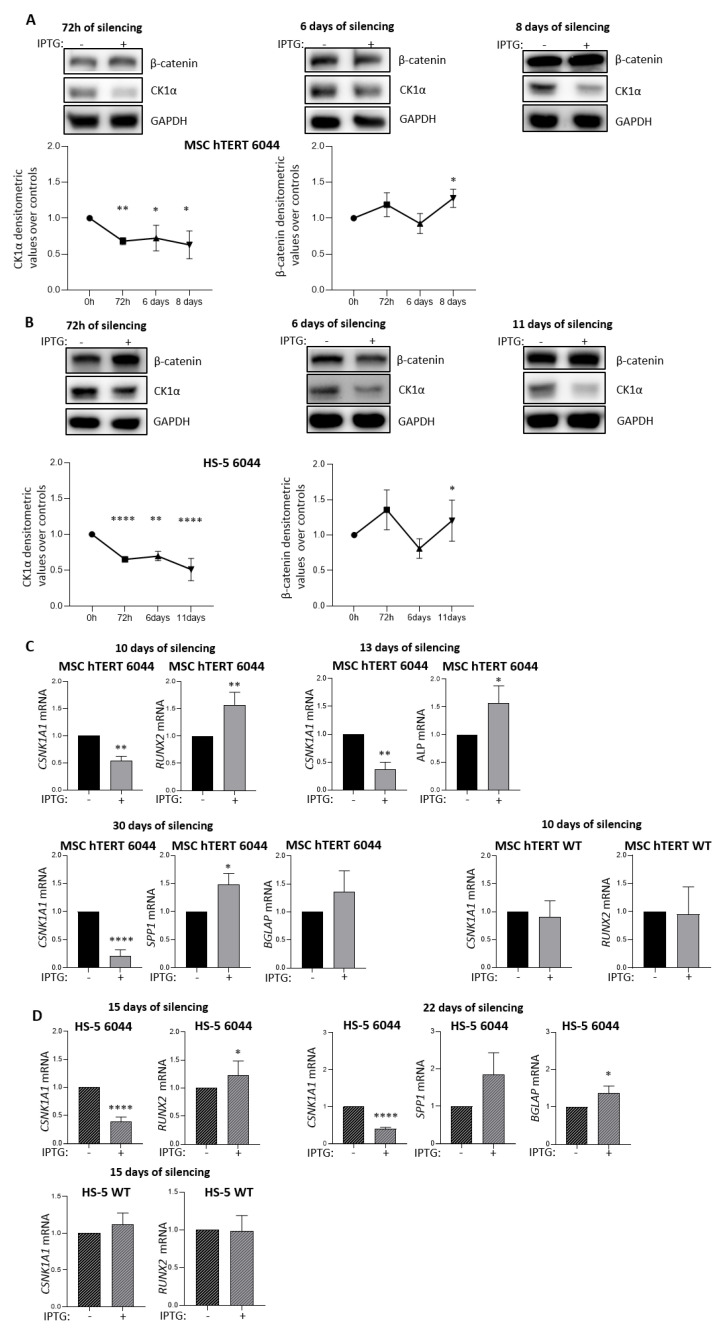
CK1α silencing modulates the expression of osteogenic differentiation markers and β-catenin in stromal cells. Protein expression of CK1α and β-catenin in MSC-hTERT 6044 cells (**A**) and in HS-5 6044 cells (**B**) treated with IPTG 500 µM over time. The different time points are indicated in each panel. The figure shows the representative WBs (upper panel) and densitometric analyses (lower panel) of CK1α/GAPDH and β-catenin/GAPDH expressed as arbitrary units over untreated cells at the corresponding time points (72 h, 6 days, 8 days for MSC-hTERT 6044 cells and 72 h, 6 days, 11 days for HS-5 6044 cells) indicated as the means ± SDs of n = 3 independent experiments for each time point for both MSC lines (**A**/**B**)). qRT-PCR analysis of *CSNK1A1*, *RUNX2*, *ALP*, *SPP1* and *BGLAP* mRNA in MSC-hTERT 6044 cells (**C**) and of *CSNK1A1*, *RUNX2*, *SPP1* and *BGLAP* mRNA in HS-5 6044 cells (**D**) treated with IPTG 500 µM over time. The different time points of CK1α silencing are indicated in each panel. Data for MSC-hTERT 6044 cells are presented as the means ± SDs of n = 8 independent experiments for *RUNX2* and *CSNK1A1* ((**C**), left), of n = 3 independent experiments for *CSNK1A1* and *ALP* ((**C**), right) and of n = 3 independent experiments for *CSNK1A1*, *SPP1* and *BGLAP* ((**C**), lower). Data for HS-5 6044 cells are presented as the means ± SDs of n = 6 independent experiments for *CSNK1A1* and *RUNX2* ((**D**), left) and of n = 3 independent experiments for *SPP1* and *BGLAP* ((**D**), right). Analysis of *CSNK1A1* and *RUNX2* after 10 days of IPTG 500 µM treatment in MSC-hTERT WT cells ((**C**), right panel) and after 15 days in HS5-WT cells ((**D**), lower panel), performed as a control. Data are presented as the means ± SDs of n = 3 independent experiments for both cell lines. *GAPDH* was used as a housekeeping gene. * *p* < 0.05, ** *p* < 0.01, **** *p* < 0.0001 compared to untreated cells. Full pictures of the Western blots are presented in Figure S2.

The Wnt/β-catenin pathway plays a pivotal role in the early phases of MSC osteogenic differentiation through *RUNX2* gene expression [12], and CK1α negatively regulates β-catenin activity [22]. Therefore, we investigated whether *CSNK1A1* gene silencing could modulate the osteogenic differentiation of MSCs through β-catenin stabilization. We used both MSC-hTERT and HS-5 stromal cell lines to generate two *CSNK1A1* shRNA IPTG-inducible stromal cell clones called, respectively, MSC-hTERT 6044 and HS-5 6044. As expected, CK1α silencing induced β-catenin accumulation in both cell lines, as observed via immunoblot analysis, which revealed β-catenin increase after 72 h and 8-11 days of CK1α silencing in MSC-hTERT and HS-5 cells (Figure 1A,B). Subsequently, we analyzed the levels of osteogenic differentiation gene markers, such as *RUNX2*, *ALP*, *SPP1* and *BGLAP*, at different time points after CK1α silencing. We treated MSC-hTERT 6044 cells with IPTG 500 μM for up to 30 days and HS-5 6044 cells for up to 22 days. 

We confirmed the persistence of *CSNK1A1* silencing at each time point and, as expected, the upregulation of the different osteogenic markers both in MSC-hTERT shRNA 6044 (Figure 1C) and in HS-5 shRNA 6044 cells (Figure 1D). To exclude off-target effects of IPTG *per se* and confirm the specificity of the CK1α silencing method used, we treated both MSC-hTERT WT (Figure 1C) and HS-5 WT (Figure 1D) cells with IPTG 500 μM and observed no significant changes in either *CSNK1A1* or *RUNX2* expression, corroborating the selectivity of the silencing strategy.

### 3.2. Activation of the Wnt/β-Catenin Signaling Pathway Is Associated with the Upregulation of RUNX2 Expression in Stromal Cells

**Figure 2:**
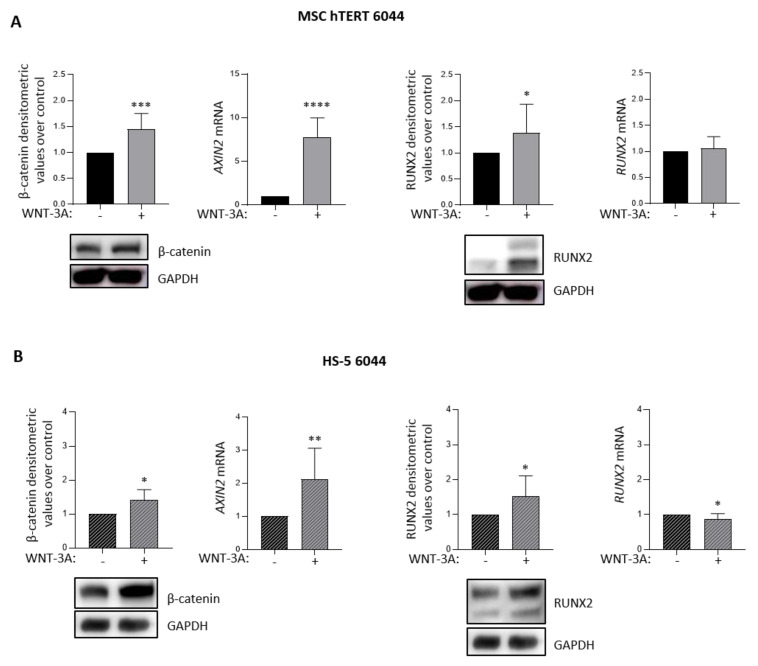
Wnt/β-catenin pathway stimulation increases RUNX2 expression in MSC cell lines. Protein expression of β-catenin and RUNX2 in MSC cell lines MSC-hTERT 6044 (**A**) and HS-5 6044 (**B**) treated with Wnt-3A 200 ng/mL for 8h (**A**) and 4h (**B**). GAPDH was used as a loading control. The figure shows the representative WBs and densitometric analyses of β-catenin/GAPDH and RUNX2/GAPDH expressed as arbitrary units over untreated cells (means ± SDs) of n = 10 independent experiments for MSC-hTERT 6044 (**A**) and n = 7 independent experiments for HS-5 6044 (**B**) cells. qRT-PCR analysis of *AXIN2* ((**A**/**B**), middle) and *RUNX2* mRNA ((**A**/**B**), right) in MSC-hTERT 6044 cells (**A**) and in HS-5 6044 cells (**B**) treated with Wnt-3A at the same concentrations for the times reported above. *GAPDH* was used as a housekeeping gene. Data are presented as the means ± SDs of n = 9 independent experiments for MSC-hTERT 6044 cells and n = 7 independent experiments for HS-5 6044 cells. * *p* < 0.05, ** *p* < 0.01, *** *p* < 0.001, **** *p* < 0.0001 compared to untreated cells. Full pictures of the Western blots are presented in Figure S2.

Considering the pivotal role of the Wnt/β-catenin pathway in the control of RUNX2 expression in other cell models, such as mouse embryonic fibroblasts and osteoprogenitor cells [19], we investigated the Wnt/β-catenin/RUNX2 axis, also, in our experimental models. We stimulated MSC-hTERT 6044 and HS-5 6044 cells with 200 ng/mL of recombinant Wnt-3A protein for 8 h and 4 h, respectively, and analyzed RUNX2 expression. We confirmed Wnt/β-catenin cascade activation in both MSC cell lines, since the expression of β-catenin and of its downstream transcriptional target *AXIN2* were found to be increased (Figure 2). Activation of Wnt signaling also caused an increase in RUNX2 protein expression in both MSC-hTERT and HS-5 cells. Therefore, the Wnt/β-catenin pathway leads to an increase in RUNX2 expression, also, in our experimental model. 

### 3.3. The Role of CK1α in Plasma Cell–Stromal Cell Cross-Talk in a Bone Marrow Microenvironment Model 

**Figure 3:**
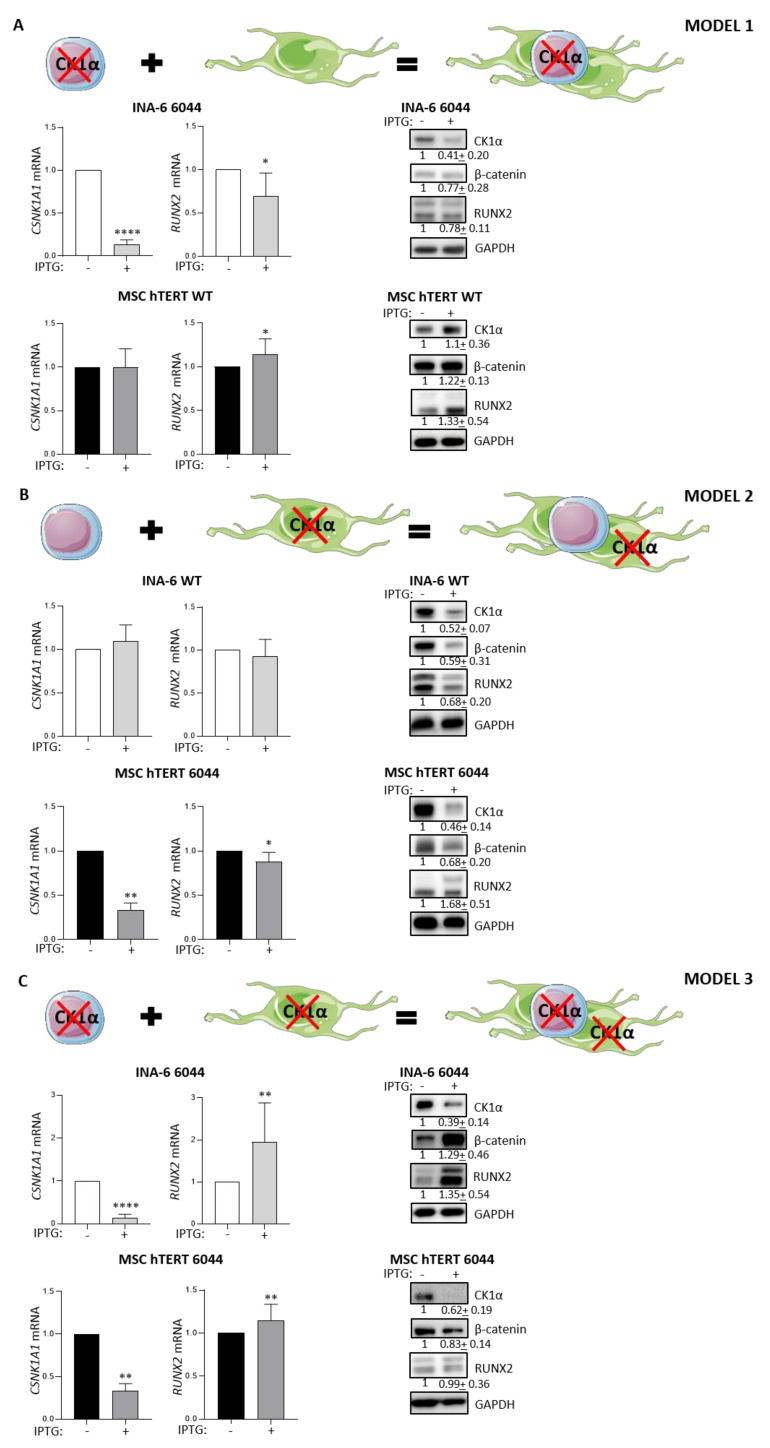
Role of CK1α in plasma cell–stromal cell cross-talk in bone marrow microenvironmental models. Upper panels: schematic representations of the experimental design. The figure was drawn using Servier Medical art templates licensed under a Creative Commons Attribution 3.0 Generic License (http://smart.servier.com/; accessed on 9 January 2022). (**A**) qRT-PCR analysis of *CSNK1A1* and *RUNX2* mRNA in both INA-6 6044 (middle panel) and MSC-hTERT WT cells (lower panel). To silence CK1α, INA-6 6044 cells were treated with IPTG 500 μM for 1 week and subsequently grown on a feeder layer of MSC-hTERT WT cells in the continuous presence of IPTG for an additional 3 days. Data are presented as the means ± SDs of at least n = 6 independent experiments. *GAPDH* was used as a housekeeping gene. The right panel shows the representative WBs and averaged densitometric analyses of CK1α/GAPDH, β-catenin/GAPDH and RUNX2/GAPDH proteins expressed as arbitrary units over untreated cells (means ± SDs) of at least n = 6 independent experiments. GAPDH was used as a loading control. (**B**) qRT-PCR analysis of *CSNK1A1* and *RUNX2* mRNA in CK1α-proficient INA-6 WT (middle panel) and CK1α-deficient MSC-hTERT 6044 cells (lower panel). INA-6 WT cells were co-cultured with CK1α-deficient MSC-hTERT 6044 cells (silenced for *CSNK1A1* by treatment with IPTG 500 μM for 1 week and subsequently used as a feeder layer for INA-6 WT in the continuous presence of IPTG for an additional 3 days). Data are presented as the means ± SDs of n = 6 independent experiments. On the right, the panel shows the representative WBs and averaged densitometric analyses of CK1α/GAPDH, β-catenin/GAPDH and RUNX2/GAPDH proteins expressed as arbitrary units over untreated cells (means ± SDs) of n = 4 independent experiments. GAPDH was used as a loading control. (**C**) qRT-PCR analysis of *CSNK1A1* and *RUNX2* mRNA in CK1α-deficient INA-6 6044 cells co-cultured with CK1α-deficient MSC-hTERT 6044 cells. Both INA-6 6044 and MSC-hTERT 6044 cells were silenced for *CSNK1A1* by treatment with IPTG 500 μM for 1 week and subsequently co-cultured in the continuous presence of IPTG for an additional 3 days. Data are presented as the means ± SDs of n = 13 independent experiments. On the right, the figure displays the representative WBs and averaged densitometric analyses of CK1α/GAPDH, β-catenin/GAPDH and RUNX2/GAPDH proteins expressed as arbitrary units over untreated cells (means ± SDs) of at least n = 7 independent experiments. GAPDH was used as a loading control. * *p* < 0.05, ** *p* < 0.01, **** *p* < 0.0001 compared to untreated cells. Full pictures of the Western blots are presented in Figure S2.

To explore whether CK1α could regulate osteoblastogenesis in the context of MMABD, we modeled a BM TME by co-culturing IL-6-dependent INA-6 cells and the stromal cell line MSC-hTERT. In particular, we created, for the first time, three different novel co-culture setups, in which CK1α silencing was achieved in MM cells (model 1), in MSC cells (model 2) and in both cell populations (model 3). Since RUNX2 expression depends on Wnt/β-catenin [19] and CK1α regulates this pathway, we also asked whether CK1α silencing could alter RUNX2 expression both in MM cells and/or in the stromal counterparts. A schematic representation of these experimental models is depicted in Figure 3. 

In the first co-culture model, we analyzed the effects of CK1α silencing on MM cell survival and RUNX2 expression in MM and stromal cells. The analysis, performed on MSC and MM cells sorted by fluorescence-activated cell sorting (FACS) exploiting GFP expression in MSC-hTERT, revealed that *RUNX2* mRNA and RUNX2 and β-catenin protein expression levels in INA-6 6044 cells were positively correlated with CK1α levels, since they were substantially reduced in the CK1α-silenced INA-6 6044 cells (Figure 3A, middle panel). Surprisingly, in MSC-hTERT WT cells, *RUNX2* mRNA and RUNX2 and β-catenin protein levels were increased (Figure 3A, lower panel). 

In the second co-culture model, CK1α-proficient INA-6 WT cells were cultured on a feeder layer of CK1α-deficient MSC-hTERT 6044 cells previously treated for seven days with IPTG 500 μM to knock down CK1α kinase. The silencing was maintained for an additional 3 days in the co-culture, at the end of which the harvest and the analysis were performed as described above. In this model, as expected, we did not observe any changes in *CSNK1A1* mRNA expression in the MM compartment INA-6 WT cells, nor, as expected, any transcriptional variation in *RUNX2* mRNA level gene expression (Figure 3B, middle panel). Unexpectedly, CK1α protein expression in INA-6 WT cells was decreased by about 50%. Accordingly, both β-catenin and RUNX2 protein levels were reduced (Figure 3B, middle panel), analogously to what was observed in model 1, in which CK1α silencing in MM cells led to decreased β-catenin and RUNX2 (Figure 3A, middle panel). Focusing on the stromal compartment in model 2, we confirmed a reduction in CK1α levels of about 66% at the mRNA level and 54% at the protein level (Figure 3B, lower panel). *RUNX2* mRNA levels were slightly but significantly reduced by 12%, but, surprisingly, RUNX2 protein levels were increased by about 68% compared to untreated cells. β-catenin protein levels were found, however, to be reduced by about 32% (Figure 3B, lower panel). 

We next asked whether the reductions in β-catenin and RUNX2 protein expression in INA-6 cells in models 1 and 2 could be associated with a higher PC death rate. Indeed, by examining the amount of apoptosis in MM cells in these two models, we observed that CK1α silencing in PCs or in stromal cells grown in co-culture led to increased PC death.

In the third co-culture model, both INA-6 6044 and MSC-hTERT 6044 cells were CK1α-silenced. 

*CSNK1A1* silencing was efficiently obtained both in INA-6 6044 cells and in MSC-hTERT 6044 cells, with reductions in mRNA levels of about 86% and 67%, respectively. Unexpectedly, β-catenin was increased in CK1α-silenced INA-6 6044 cells and it was associated with augmented RUNX2 expression both at the mRNA and at the protein levels (Figure 3C, middle panel). In the stromal counterparts, *RUNX2* mRNA expression was mildly but significantly increased, but this increment did not translate into RUNX2 protein upregulation. A slight decrease in β-catenin expression was instead observed upon CK1α silencing in the MSC-hTERT cells (Figure 3C, lower panel). 

As a control, a co-culture model of INA-6 WT and MSC-hTERT WT cells was performed, with no statistically significant differences in CK1α or RUNX2 expression being found either in MM compartments or MSC cells, confirming the specificity of the observed changes (Figure S1). 

### 3.4. Cell Adhesion Sustains RUNX2 Expression in MM Cells 

**Figure 4:**
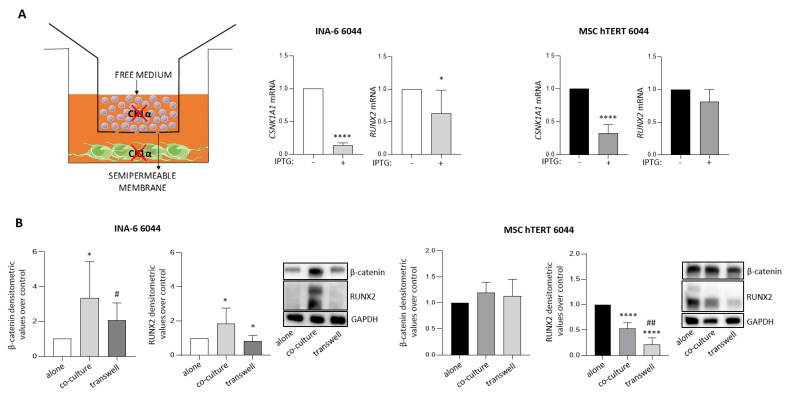
(**A**) Left panel: schematic representation of the experimental design. Right panel: qRT-PCR analysis of *CSNK1A1* and *RUNX2* mRNA in INA-6 6044 and MSC-hTERT 6044 cells, both silenced for *CSNK1A1* with IPTG 500 μM for 1 week and subsequently co-cultured in the continuous presence of IPTG for an additional 3 days using a transwell system. Data are presented as the means ± SDs of at least n = 5 independent experiments. *GAPDH* was used as a housekeeping gene. (**B**) Densitometric analyses (left panel) and representative WBs (right panel) of RUNX2/GAPDH and β-catenin/GAPDH basal protein expression in alone/co-culture/transwell conditions, both in INA-6 6044 cells (left panel) and in MSC-hTERT 6044 counterparts (right panel). Protein expression data are presented as the means ± SDs of n = 5 independent experiments. * *p* < 0.05, **** *p* < 0.0001 compared to the alone condition; # *p* < 0.05, ## *p* < 0.01 compared to the co-culture condition. Full pictures of the Western blots are presented in Figure S2.

To investigate more in depth the role of CK1α silencing in the third co-culture model and to explain the unexpected elevation in RUNX2 levels in PCs after co-silencing CK1α in MM and MSC-hTERT cells (Figure 3, model 3), we modified this third model by physically separating MM and MSC cells using a transwell system. As reported in Figure 4A, CK1α silencing in MM cells and in stromal compartments was efficient. Surprisingly, in these conditions, *RUNX2* gene expression was significantly decreased in PCs, in contrast to its upregulation observed when cell–cell contact was preserved (Figure 3C). Moreover, *RUNX2* mRNA expression in MSCs was unchanged (Figure 4A, right panel), with a different trend observed compared to the data shown in Figure 3C. 

These results indicate that cell–cell interactions established in the BM niche are critical for the control of RUNX2 expression both in MM cells and in MSC cells. Moreover, these results suggest that fluctuations in RUNX2 expression in MM cells are somehow associated with substantial changes in its expression in the stromal compartment, also, as indicated by the observation of an increase in its expression in MSC cells when it was downregulated in MM cells (models 1 and 2). 

To further investigate the role of soluble factors or cell–cell contacts in the modulation of RUNX2 expression, we compared basal RUNX2 expression in MSC and MM cells in different experimental conditions: (1) alone: MM cells grown without a MSC feeder layer and MSCs grown without MM cells; (2) co-culture: MM cells grown on a feeder layer of MSCs, in which the cross-talk was mediated both by soluble factors and by cell–cell interactions; (3) transwell co-culture: MM cells and MSCs grown in co-culture but physically separated so that cross-talk was mediated only by soluble factors. 

Interestingly, in condition 2 “co-culture” of INA-6 with MSC-hTERT cells, both β-catenin and RUNX2 protein expression increased in the MM cell population, at variance with condition 1, “alone” (Figure 4B, left panel). In condition 3 “transwell co-culture”, RUNX2 expression in PCs was comparable to that seen in condition 1 (Figure 4B, left panel). On the contrary, RUNX2 expression in the MSC-hTERT population was lower in condition 2 compared to condition 1, and it was further reduced when grown in condition 3 (Figure 4B, right panel). In MSCs, no significant changes were observed in β-catenin protein expression across all the experimental conditions (Figure 4B, right panel). 

### 3.5. The Role of Lenalidomide in Stromal Cell Osteogenic Differentiation Potential

**Figure 5:**
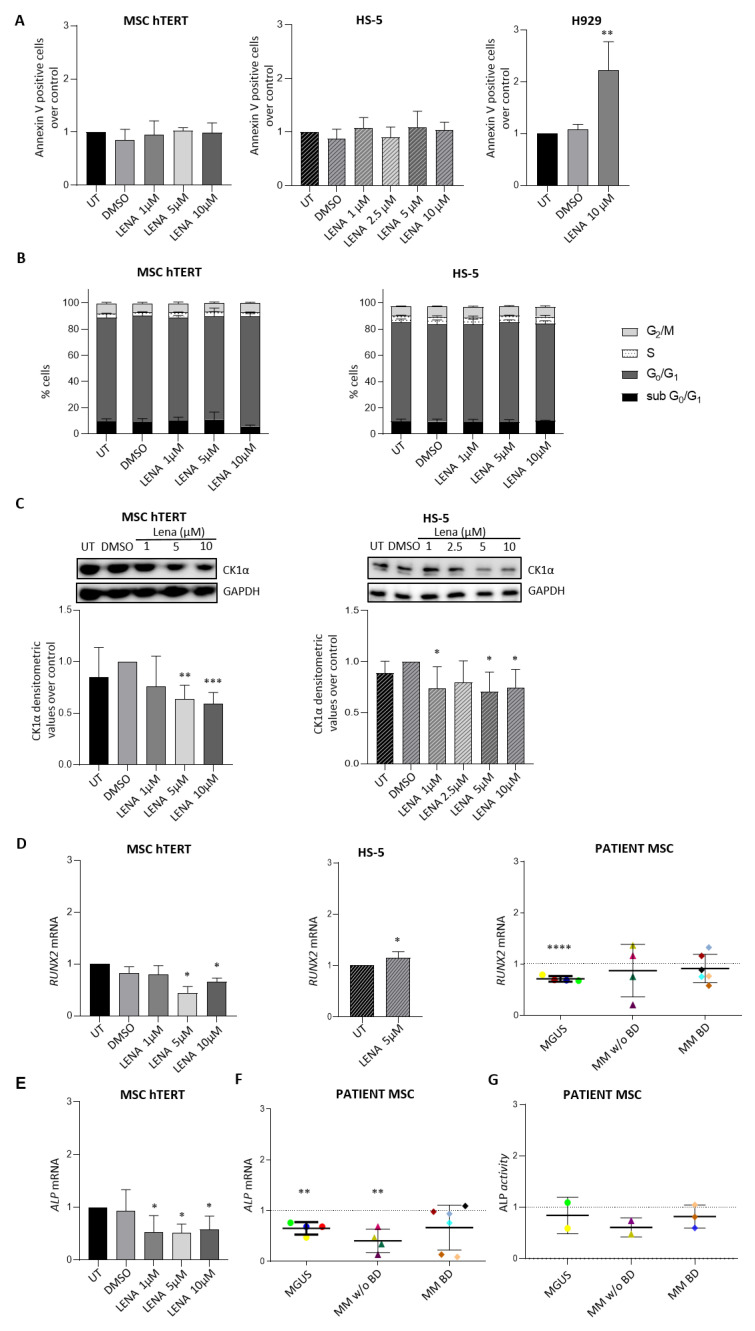
Lenalidomide induces CK1α degradation and negatively modulates osteogenic differentiation markers in stromal cells. (**A**) Quantification of apoptosis through Annexin V staining and FACS analysis in MSC-hTERT (left panel) and HS-5 (middle panel) cell lines treated with different concentrations of Lena (ranging from 1 to 10 µM) for 7 days. H929 MM cells were used as a positive control for Lena efficacy (right panel). Data are normalized over untreated (UT) cells and are presented as the means ± SDs of n = 4 independent experiments for both MSC-hTERT cells and H929 cells, and of n = 8 independent experiments for HS-5 cells. DMSO 0.002% V/V was tested to exclude vehicle-induced toxicity. ** *p* < 0.01 compared to UT cells (**B**) Quantification of cell cycle phases through PI staining and FACS analysis in MSC-hTERT (left panel) and HS-5 cells (right panel) after treatment with Lena at different concentrations (1, 5, 10 µM) for 7 days. Data are presented as the means ± SDs of n = 4 independent experiments for MSC-hTERT cells and of n = 6 independent experiments for HS-5 cells. (**C**) Representative WBs (upper panel) and averaged densitometric analyses (lower panel) of CK1α/GAPDH expression after Lena treatment (ranging from 1 to 10 µM) for 7 days in MSC-hTERT cells (left panel) and in HS-5 cells (right panel). Data are normalized over DMSO-treated cells and are presented as the means ± SDs of n = 4 independent experiments. DMSO 0.002% V/V was tested to exclude vehicle-induced toxicity. * *p* < 0.05, ** *p* < 0.01, *** *p* < 0.001 compared to the DMSO condition. (**D**) qRT-PCR of *RUNX2* mRNA expression in MSC-hTERT cells (left panel), HS-5 cells (middle panel) and patient-derived MSCs (dispersion graph, right panel) with MGUS, MM without bone disease (MM w/o BD) or affected by bone disease (MM BD) and treated with Lena 2.5 µM for 7 days. Each patient is represented by a different color. *GAPDH* was used as a housekeeping gene. Data are normalized over untreated (UT) cells and are presented as the means ± SDs of n = 4 independent experiments for MSC-hTERT cells (treated for 7 days) and of n = 5 independent experiments for HS-5 cells (treated for 3 days). * *p* < 0.05, **** *p* < 0.0001 compared to the UT condition. In the *RUNX2* mRNA dispersion graph, data were normalized to the *GAPDH* housekeeping gene and compared with the DMSO condition, indicated by the dotted line. DMSO 0.005% V/V was used to confirm the non-toxicity of the vehicle. **** *p* < 0.0001 compared to the DMSO condition. (**E**) qRT-PCR of *ALP* mRNA expression in MSC-hTERT cells treated with Lena 1, 5, 10 µM for 7 days. Data are presented as the means ± SDs of n = 4 independent experiments and were compared with the UT condition. *GAPDH* was used as a reference gene. DMSO 0.002% V/V was used to confirm the non-toxicity of the vehicle. * *p* < 0.05 compared to the UT condition. (**F**) Dispersion graph of *ALP* mRNA expression quantified by qRT-PCR in patient-derived MSCs (left) treated with Lena 2.5 µM for 7 days. DMSO 0.005% V/V was used to confirm the non-toxicity of the vehicle. Each patient is represented by a different color. Data were normalized to the *GAPDH* housekeeping gene and compared with the DMSO condition, indicated by the dotted line. ** *p* < 0.01 compared to the DMSO condition. (**G**) Dispersion graph of ALP-secretion activity quantified using a colorimetric ALP assay performed on the supernatants of patient-derived MSCs treated with DMSO or Lena 2.5 µM for 7 days. Each patient is represented by a different color The data were normalized to ALP activity in the DMSO condition, indicated by the dotted line. Full pictures of the Western blots are presented in Figure S2.

To date, the effects of IMiDs, which provoke CK1α degradation, on MSC differentiation towards osteogenic lineages are still debated. To investigate the role of Lena on the osteoblastogenic potential of MSCs, we first evaluated in our experimental models whether Lena could affect MSC viability. As shown in Figure 5, treatment for seven days with different concentrations of Lena did not induce any cytotoxicity (panel A) nor any cell cycle changes (panel B) in either of the MSC lines tested. As a positive control for Lena activity, we treated with the drug the Lena-sensitive MM cell line H929 (Figure 5A) and observed an increase in apoptosis upon treatment.

Considering that Lena induces CK1α proteasomal degradation in MM cells [23], we treated MSC-hTERT and HS-5 cells with different concentrations of Lena for seven days and found that CK1α was also significantly reduced in these cells (Figure 5C), as previously shown in [41] for MSC-hTERT cells.

Surprisingly, Lena treatment was associated with a significant reduction in *RUNX2* mRNA levels in MSC-hTERT cells (Figure 5D, left panel), as opposed to an increase in HS-5 cells (Figure 5D, middle panel). We also treated primary BMSCs collected from BM biopsies of patients affected by MGUS and MM with or without bone disease (BD) (Table 1). BMSCs were treated with Lena 2.5 μM for seven days. A significant reduction in *RUNX2* was detected in cells from MGUS patients and a decreasing trend was observed in cells of four out of six samples of MM with BD (Figure 5D, right panel). Upon Lena treatment, *ALP* mRNA expression was found to be reduced in MSC-hTERT cells (Figure 5E) and in primary BMSCs from MGUS or MM patients without BD with a trend towards reduction in MM with BD (Figure 5F). Measurement of ALP enzymatic activity in some supernatants of primary Lena-treated BMSCs revealed a trend towards reduction at each stage of the disease (Figure 5G), with the exception of the MGUS stage, for which sufficient samples were not available for the analysis.

**Figure 6:**
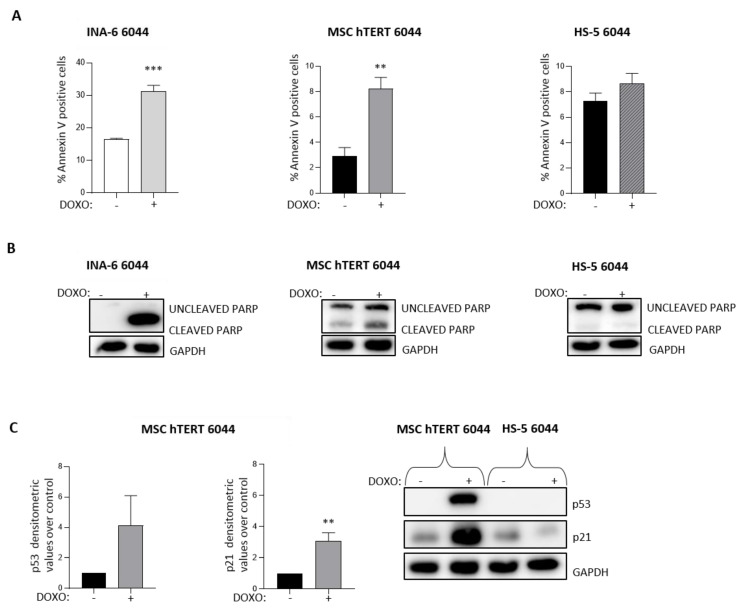
Doxorubicin treatment modulates cell apoptosis differently in MSC-hTERT and HS-5 cell lines. (**A**) Quantification of apoptosis through Annexin V staining and FACS analysis in INA-6 6044 (left panel), MSC-hTERT 6044 (middle panel) and HS-5 6044 (right panel) cells treated with 1.2 μM Doxorubicin (Doxo) for 18h. Data are presented as the means ± SDs of n = 3 independent experiments. (**B**) Representative WBs of PARP cleavage expression after Doxo treatment in INA-6 6044 (left panel), MSC-hTERT 6044 (middle panel) and HS-5 6044 cells (right panel). (**C**) Densitometric analyses (left panel) and representative WBs (right panel) of p53 and p21 expression after Doxo 1.2 μM treatment for 18 h in MSC-hTERT 6044 and HS-5 6044 cells. GAPDH was used as a housekeeping protein. Data are presented as the means ± SDs of n = 3 independent experiments. ** *p* < 0.01, *** *p* < 0.001 compared to untreated cells. Full pictures of the Western blots are presented in Figure S2.

Given the opposite results caused by Lena treatment regarding RUNX2 expression in MSC-hTERT or HS-5 cells (Figure 5D), we looked for differences between the two cell lines. The immortalization method used was mediated by HPV-16 E6/E7 for HS-5 cells (ATCC bio resources and [47]) and by enforced expression of the catalytic subunit of telomerase for MSC-hTERT cells [43]. It has been reported that the *E6/E7* gene products interfere with the function of p53 and Rb1, respectively, thereby favoring cell cycle progression [48]. Considering that CK1α is a major regulator of MDM2/p53 signaling [49] and that p53 negatively regulates *RUNX2* expression [50], we asked whether p53 could be differentially activated in HS-5 or MSC-hTERT cells. We therefore treated HS-5 6044, MSC-hTERT 6044 and INA-6 6044 cells with Doxorubicin (Doxo) 1.2 μM for 18 h—a stimulus which is known to activate the p53 signature. INA-6 6044 was used as a positive control for Doxo-mediated p53 induction. As expected, Doxo led to an increased percentage of Annexin V positive cells in INA-6 6044 cultures and consequent increased apoptotic cell death rate (Figure 6A, left panel). MSC-hTERT 6044 cells showed a strong sensitivity to Doxo treatment by undergoing apoptosis (Figure 6A, middle panel), while HS-5 6044 cells showed a substantial degree of resistance to the treatment, with no significant changes in apoptosis observed upon Doxo treatment (Figure 6A, right panel). Immunoblot analysis revealed PARP cleavage only in MSC-hTERT cells (Figure 6B) and in the INA-6 6044 positive control, as well as upregulation of p53 and its downstream target p21 only in MSC-hTERT cells after Doxo treatment (Figure 6C), which suggested that the resistance of HS-5 cells to Doxo could rely on a defective p53 pathway. 

## 4. Discussion

Even if the survival of MM patients has significantly improved in the last 15 years, the disease remains incurable [51]. Most MM patients present SREs, which negatively affect their quality of life [5]. It is therefore mandatory to study the pathophysiological mechanisms that sustain MM PC expansion and perturb bone homeostasis in order to develop novel effective therapies targeting both the hematological and bone diseases. In this work, we have showed that the oncogenic kinase CK1α could not only sustain clonal MM PC growth but also regulate the osteoblastogenic potential of stromal cells. 

Protein kinase CK1α acts as a negative regulator of the Wnt/β-catenin pathway [22,52], which plays a pivotal role in the commitment of MSCs to the osteoblastic lineage [39,53]. Our data demonstrate that CK1α silencing in MSCs caused variable changes in β-catenin expression. β-catenin was not, in fact, always upregulated over time in both of the MSC cell lines used (Figure 1A,B). A negative feedback loop involving the β-catenin transcriptional target gene *AXIN2* has been demonstrated in HEK293 cells [54]. In these cells, overexpression of AXIN2 inhibited the activity of wild-type β-catenin [54]. Therefore, the oscillatory β-catenin expression observed in our models could have been caused not only exclusively by CK1α reduction but also by other mechanisms that could include *AXIN2.* Indeed, it is conceivable that the induced expression of β-catenin upon CK1α silencing is sufficient to cause an increase in AXIN2 expression, which, in turn, could decrease β-catenin expression via an autoregulatory feedback loop mechanism, as described in HEK293 cells [54]. 

We next determined that β-catenin protein upregulation induced by CK1α silencing was sufficient to sustain the upregulation of different osteogenic differentiation markers in stromal cells, such as *RUNX2* (early), *ALP*, *SPP1* and *BGLAP* (late), at different time points, both in CK1α-deficient MSC-hTERT 6044 cells and in HS-5 6044 cells (Figure 1 C/D). The strong increase in RUNX2 protein but not mRNA expression after β-catenin signaling activation through Wnt-3A stimulation confirmed the link between β-catenin and RUNX2 in our tested stromal cell models (Figure 2), previously demonstrated in other cell types [19]. 

Drissi et al.’s study revealed an auto-regulatory feedback loop involving RUNX2 in osteoblastic osteosarcoma ROS 17/2.8 cells and in the fibroblast cell line NIH3T3. In these cells, RUNX2 protein overexpression was sufficient to inhibit the activity of its own promoter, reducing its transcriptional expression [55]. Whether this putative RUNX2 autoregulation loop was present in our models, also, is currently unknown and deserves further investigation. 

We next evaluated how CK1α inactivation in a recreated in vitro BM microenvironment model could be exploited to implement the osteoblastogenic potential of MSCs. The development of different CK1α loss-of-function cell models allowed us to selectively knock down the kinase in PCs or in MSCs. By exploiting three different co-culture models of MSC and MM cells, in which CK1α silencing was achieved in the MM compartment (model 1), in the MSC compartment (model 2) and in both cell populations (model 3), we were able to finely analyze the role of CK1α in the BM microenvironment and in PC–MSC cross-talk.

Our results unexpectedly revealed, for the first time, that CK1α inactivation in the BM co-culture systems regulates RUNX2 expression both in MM cells and in MSCs, possibly through Wnt/β-catenin signaling cascade modulation. In detail, CK1α silencing in MM cells reduced both β-catenin and RUNX2 expression in PCs, potentially counteracting the MSC osteogenic differentiation block induced by malignant PCs. Indeed, RUNX2 expression, sustained by β-catenin upregulation, was found to be increased in the stromal counterpart (Figure 3A). Corroborating our data, it was previously reported that the inhibitory effect of MM cells on osteoblast differentiation appears to be mediated in part by the capability of MM cells to constrain RUNX2 activity in human MSC and osteoprogenitor cells [16]. Moreover, evidence has confirmed that RUNX2 expression and activity in MM cells sustain angiogenesis, cell survival and tumor progression, leading to poor prognosis [17]. Therefore, our data point to a novel unanticipated role for CK1α in the sustention of PC-restricted RUNX2 expression, which, in turn, could hamper the osteoblastogenic potential of stromal cells. Therefore, targeting CK1α in PCs could be beneficial not only in terms of killing MM cells, as observed in [23,37], but also in terms of downregulating the detrimental effects of MM-restricted RUNX2 protein expression. 

Surprisingly, when CK1α silencing was achieved in stromal cells in co-culture model 2, we could determine CK1α and RUNX2 protein reduction, also, in PCs (Figure 3B), leading to subsequent RUNX2 protein upregulation in the stromal counterpart. In contrast to the results obtained for model 1, for the first time, β-catenin levels did not correlate with RUNX2 levels only in CK1α-silenced stromal cells, which displayed reduced β-catenin and higher RUNX2 protein expression compared with untreated cells. As recently reviewed by K. Sweeney et al. [56], the Wnt/β-catenin pathway could control RUNX2 expression, which, in turn, might regulate not only the expression of osteoblastogenic-related genes but also a variety of Wnt ligands, TCF/LEF co-activators and Wnt inhibitors, such as Dikkopf-1 (DKK-1) [57], which could suppress Wnt signaling during human osteoblast differentiation [21]. Therefore, RUNX2 might potentially negatively modulate β-catenin signaling itself, as reported in primary osteoblastic cells, where RUNX2 overexpression led to GSK3β activation, affecting β-catenin stability [58]. Therefore, the strong upregulation of RUNX2 protein expression detected in the stromal compartment in model 2 could be sufficient to negatively regulate Wnt/β-catenin signaling components to further reduce β-catenin activity in our experimental model, also. Intriguingly, it has been shown that after initial β-catenin and RUNX2 upregulation, their prolonged overexpression negatively affects the capability of preosteoblasts to differentiate into mature osteoblasts [21,59]. Therefore, osteoblastic cells need to reduce Wnt/β-catenin signaling activation in order to fully complete the osteogenic differentiation process [56,58]. 

In the third co-culture model, we tried to reproduce in vitro a model of CK1α inactivation in the bone marrow microenvironment as close as possible to that obtainable in vivo with the administration of a putative CK1α-specific inhibitor to patients. Unexpectedly, we found that co-silencing of CK1α both in MM and in MSC-hTERT 6044 cells produced a strong upregulation of RUNX2 in MM cells (Figure 3C).

It has previously been shown that soluble factors, such as IL-7 and adhesion molecules such as Very Late Antigen-4/Vascular Cell Adhesion molecule-1 (VLA-4/VCAM-1), could regulate RUNX2 expression in a co-culture system of MM cells and BMSCs [15]. Moreover, Trotter et al. reported that the RUNX2/PI3K/AKT axis in MM cells is an important driving force in tumor progression as well as an indicator of poor prognosis [17]. The PI3K/AKT pathway could stimulate RUNX2 activity by controlling the expression of different proteins, such as SMURF2, FOXO1 and FOXO3, which are indirectly related to RUNX2 stability [60]. In turn, RUNX2 is also able to enhance PI3K/AKT signaling by upregulating p85, p110β, AKT protein levels and components of the mTORC2 complex during osteoblast and chondrocyte differentiation [60]. Therefore, the increase in RUNX2 expression in MM cells could be sustained, also, by other pathways (not only those related to the Wnt/β-catenin axis [61]), such as the PI3K/AKT cascade [60,62] or soluble factors and adhesion molecules involved in MM–MSC cross-talk, as reported in [15,63]. 

To determine whether RUNX2 upregulation could be ascribed to soluble factors, we reproduced co-culture model 3 using a transwell system in order to avoid physical interactions between the MM and stromal cell populations. In these experimental settings, the expression of RUNX2 achieved both in MM cells and in MSC counterparts (Figure 4A, right panel) contrasted with the data obtained for the maintenance of cell–cell adhesion (Figure 3C). These results highlight the importance of cell adhesion in the control of both β-catenin and RUNX2 expression. Indeed, we observed that RUNX2 and β-catenin basal expression was sustained by cell–cell interactions rather than soluble factors (Figure 4B). Moreover, soluble factors did not seem to significantly affect RUNX2 expression in PCs (Figure 4B). Very recently, it was demonstrated that RUNX2 was highly expressed in adherent B-NHL and MM cell lines compared to cells grown in suspension and that knocking down the expression of RUNX2 could overcome cell-adhesion-mediated drug resistance [62]. In MSC-hTERT cells, RUNX2 basal expression was reduced compared to its expression detected in the same cells grown alone, by co-culturing these cells with PCs both in the presence and in the absence of a transwell system, which suggested that both cell adhesion and soluble factors could be involved in the control of RUNX2 expression activity in this MSC cell line (Figure 4B). 

We and others have previously reported that Lena causes CK1α protein degradation in MM PCs [23] and in other cell types associated with hematological diseases [64]. Therefore, we analyzed the capability of Lena to modulate MSC osteblastogenic potential in view of its effect on CK1α protein expression.

In fact, while the positive effects of Lena and its derivatives on MM hematological disease are widely known, the consequences of IMiDs on MSC differentiation potential and bone remodeling are still controversial and a matter of debate [31,32,33,34,35,36]. Therefore, we investigated the effects of Lena on osteoblastogenic marker expression in our MSC models. Firstly, we proved that Lena did not cause any toxicity in the MSC lines tested (Figure 5A,B) and confirmed that it caused CK1α protein degradation in all the stromal cell lines (Figure 5C). However, it caused both *RUNX2* (Figure 5D, left) and *ALP* mRNA downmodulation (Figure 5E) in MSC-hTERT cells, while it induced *RUNX2* mRNA upregulation in HS-5 cells (Figure 5D, middle). The results obtained for BMSC patient cells suggested that the MSC-hTERT cell line could better mimic disease-related stromal cells, since trends of reduction in both RUNX2 (Figure 5D) and ALP expression levels (Figure 5F) were observed in primary BMSCs. These data indicate a negative role for Lena in the sustention of the osteoblastogenic potential of stromal cells and a consequent likely negative effect on MMABD. In support of these conclusions, it was previously reported that high secretion levels of activin A, a TGF-β family member supporting MM-induced osteolysis, were identified after Lena treatment of MM-derived BMSCs [33]. The increased activin A secretion was abrogated by the addition of an activin A-neutralizing antibody, which effectively restored osteoblastic function [33]. Moreover, RUNX2 and DLX5 expression, ALP activity and matrix mineralization were reduced after Lena treatment of human BMSC TERT+ cells in in vitro experiments, while beta A, DKK-1, gremlin 1 and activin A molecules were upregulated, indicating that Lena has a negative effect on osteoblastogenesis [31]. 

Considering that Lena treatment led to opposite results for RUNX2 expression in MSC-hTERT and HS-5 stromal cells, we suggested that this discrepancy could have been due to the different immortalization methods used for the two cell lines. The HS-5 method of immortalization consists of a LXSN-16 E6E7 retrovirus infection, which leads to p53 degradation [47,48], while the MSC-hTERT immortalization method is based on the enforced expression of the telomerase catalytic subunit [43]. Previous in vivo and in vitro experiments demonstrated that p53 function negatively modulates RUNX2 expression, regulating osteogenic differentiation [50,65,66]. Our data showed that only MSC-hTERT cells were responsive to the p53-inducing agent Doxorubicin, which actively induced a strong increase in cell apoptosis and enhanced protein expression of p53 and p21 (Figure 6). However, no significant changes in cell apoptosis or in the expression levels of p53 and its target p21 were detected in HS-5 cells, confirming a possible p53 inactivation that could be induced by the immortalization method (Figure 6). 

CK1α negatively regulates p53 function [23], which, in turn, inhibits RUNX2 expression [50]. Therefore, the reduced *RUNX2* mRNA levels observed in MSC-hTERT cells treated with Lena could depend, also, on an active p53 induction sustained by Lena through CK1α degradation in these cells. The data obtained for MSC-hTERT cells better reproduced the findings on patient-derived BMSCs, in which a reduction in osteoblastogenic marker expression was detected upon Lena treatment. Therefore, from a translational point of view, MSC-hTERT cells could represent the best model of stromal cells for use in in vitro experiments to mimic disease-related MCS.

## 5. Conclusions

Our data suggest that protein kinase CK1α is a key regulator of MM pathophysiology, since not only does it support the survival of malignant PCs through the regulation of pro-survival signaling cascades, such as β-catenin and AKT [23], but it also sustains their cross-talk with the microenvironment. We discovered an unprecedented role for CK1α in the modulation of RUNX2 expression, which differs among the type of cells tested (PCs or MSCs). Selective inhibition of CK1α in MM cells (model 1) and in MSC counterparts (model 2) in the microenvironment could decrease β-catenin and RUNX2 expression in PCs, removing the inhibitory effect of PC-restricted RUNX2 expression [15,16] and increasing PC death. On the other hand, it could support the osteogenic transcriptional program of stromal cells, mainly through MSC-dependent RUNX2 upregulation, with the potential to ameliorate MMABD. Our data showed that the CK1α/RUNX2 axis is relevant to both MM hematological and bone diseases. Therefore, they are particularly meaningful for the future design of a CK1α isoform-specific inhibitor that could be tested in a therapeutic perspective against MM plasma cellular disease and the adverse effects of MM-associated bone disease. Targeting CK1α in PCs or in stromal cells could be beneficial by reducing plasma cell clonal expansion on the one hand and promoting the recovery of bone formation on the other. The use of immunoconjugated drugs targeting the B-Cell Maturation Antigen (BCMA) is currently being investigated in clinical trials with refractory/relapsed MM patients [67]. Recently, the monoclonal antibody anti-BCMA conjugated with the MonoMethyl Auristatin F, GSK2857916 (Belantamab Mafodotin) [68], has shown promise when tested in different studies, either as a single agent or in combination with other drugs, in the therapy of heavily pre-treated MM patients [69,70]. 

Therefore, our findings on CK1α could pave the way for the future development of an anti-BCMA molecule linked (via a non-cleavable linker) to a novel, as yet to be designed specific CK1α inhibitor in order to selectively kill malignant PCs and, in parallel, sustain an osteoblastic transcriptional program in the stromal compartment, potentially ameliorating MMABD. 

Targeting the CK1α/RUNX2 axis could produce important advancements in the establishment of a better cure for MM patients.

## Figures and Tables

**Table 1:** Clinical features of patients analyzed.

MM#	DIAGNOSIS	SEX	AGE	ISS	R-ISS	PARAPROTEIN	PCs (%)	KARYOTYPE	BD	LENA	R/ND
1	MM	F	72	I	I	IgG/λ	20	monosomy 13	N	N	ND
2	MM	F	41	I	I	IgG/κ	50	gain(1q)	Y	N	ND
3	MM	F	56	I	I	IgA/κ	80	standard	N	N	R
4	MM	F	78	I	I	IgA/κ	80	t(11;14), gain(1q)	N	N	ND
5	MM	F	69	I	I	IgA/λ	52	hyperdiploidy	Y	N	R
6	MM	M	82	II	II	IgG/λ	35	del(17)	Y	N	ND
7	MM	F	71	III	III	IgG/κ	100	t(4;14), gain(1q)	Y	N	R
8	MM	F	69	I	II	IgA/κ	30	del(17p), gain1q)	N	N	ND
9	MGUS	M	83			κ	6	nd	N	N	ND
10	MGUS	M	56			IgG/κ	5	nd	N	N	ND
11	MGUS	M	49			IgG/κ	6	nd	N	N	ND
12	MM	M	67	I	I	IgG/κ	20	less of Y	Y	N	R
13	MM	M	71	I	II	IgG/κ	30	t(4;14), gain1q	Y	N	ND
14	MGUS	F	50			IgG/κ	<1	t(14;16)	N	N	ND
15	MM	M	60	III	III	κ	70	t(11;14)	Y	N	ND

MM: multiple myeloma; MGUS: monoclonal gammopathy of undetermined significance; F: female, M: male; ISS: International Staging System; R-ISS: Revised International Staging System; Ig: immunoglobulin; PCs: plasma cells; nd: not determined; BD: bone disease; N: no/absent; Y: yes/present; LENA: Lenalidomide; R: relapse; ND: new diagnosis. All features are reported as data at diagnosis.

## Data Availability

The data presented in this study are available in the article and Supplementary Materials.

## Supplementary Materials

The following supporting information can be downloaded at: https://www.mdpi.com/article/10.3390/cancers14174173/s1, Figure S1: IPTG treatment of WT INA-6–MSC-hTERT co-cultured cells did not affect RUNX2 expression, Figure S2: Original blots relative to the Western blotting analyses.
