# Supplementary material for: CK1α/RUNX2 Axis in the Bone Marrow Microenvironment: A Novel Therapeutic Target in Multiple Myeloma
Source: Cancers (Basel). 2022 Aug 29;14(17):4173. doi: 10.3390/cancers14174173 (PMC9454895; doi:10.3390/cancers14174173)
Supplement: Supplementary file 1 [file cancers-14-04173-s001.zip › cancers-1828303-supplementary.pdf]

cancers

Supplementary materials

CK1α/RUNX2 Axis in the Bone Marrow Microenvironment: A Novel Therapeutic Target in Multiple Myeloma

Anna Fregnani, Lara Saggin, Ketty Gianesin, Laura Quotti Tubi, Marco Carraro, Gregorio Barilà, Greta Scapinello, Giorgia Bonetto, Maria Pesavento, Tamara Berno, Antonio Branca, Carmela Gurrieri, Renato Zambello, Gianpietro Semenzato, Livio Trentin, Sabrina Manni and Francesco Piazza

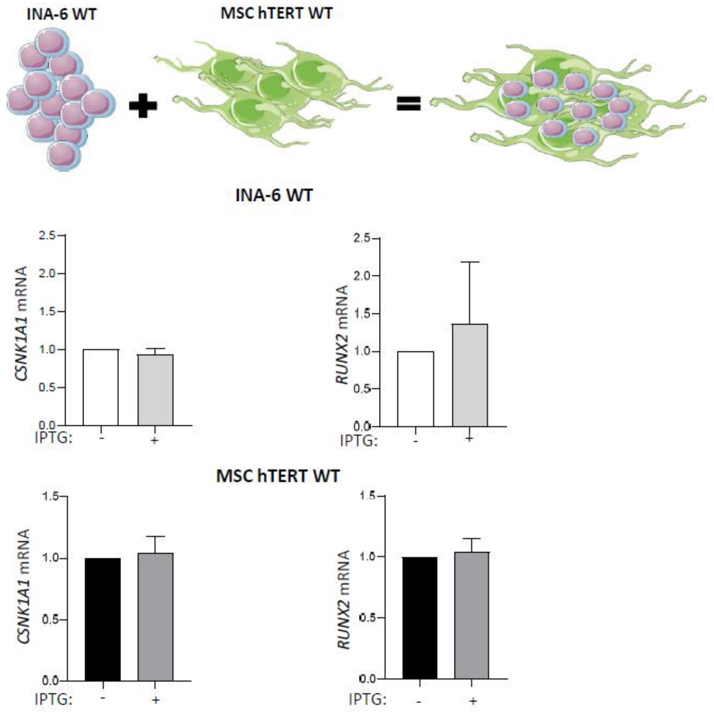

Figure S1. IPTG treatment of WT INA-6–MSC-hTERT co-cultured cells did not affect RUNX2 expression, Upper panel: schematic representation of the experimental design. The figure was drawn using Servier Medical art templates licensed under a Creative Common Attribution 3.0 Generic License. http://smart.servier.com/ accessed on 09/01/2022. qRT-PCR analysis of CSNK1A1 and RUNX2 mRNA in both INA-6 WT (middle panel) and in MSC hTERT WT cells (lower panel). CK1α proficient INA-6 WT cells were treated with IPTG 500µM for 1 week and subsequently grown on a feeder layer of CK1α proficient MSC hTERT WT in the continuous presence of IPTG for additional 3 days. Data represent mean±SD of n=3 independent experiments. GAPDH was used as housekeeping gene.

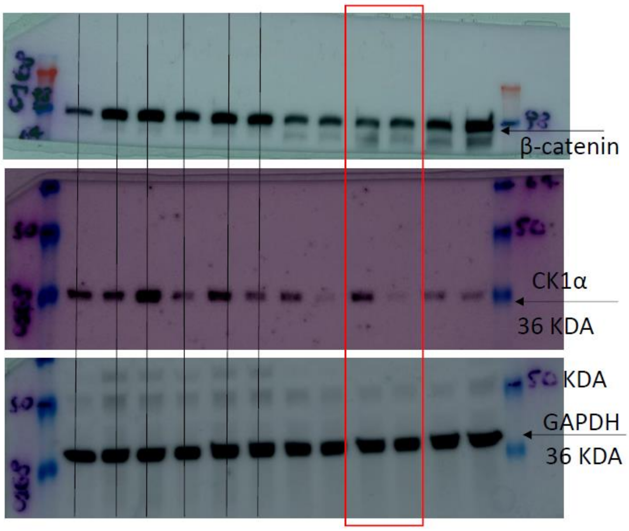

Fig. 1A
72h

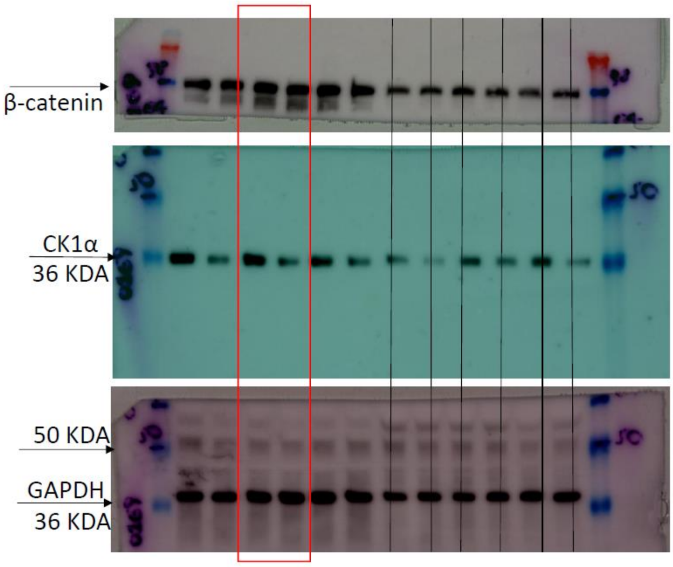

Fig. 1A
6 days

Deleted black lanes are related to experiments not presented in this manuscript. Red rectangle indicates the representative WB used for the figure, in an experiment replicated 3 times.

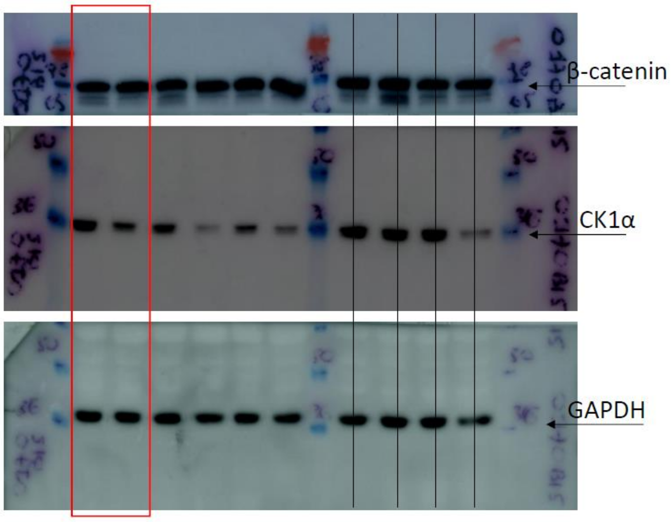

Fig. 1A
8 days

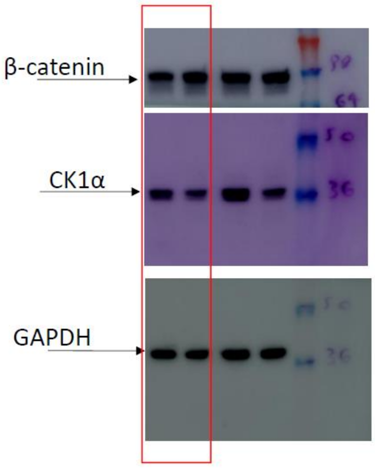

Fig. 1B
72h

Deleted black lanes are related to experiments not presented in the manuscript. Red rectangle indicates the representative WB used for the figure, in an experiment replicated 3 times for FIG.1A and in an experimenti replicated 2 times for FIG.1B.

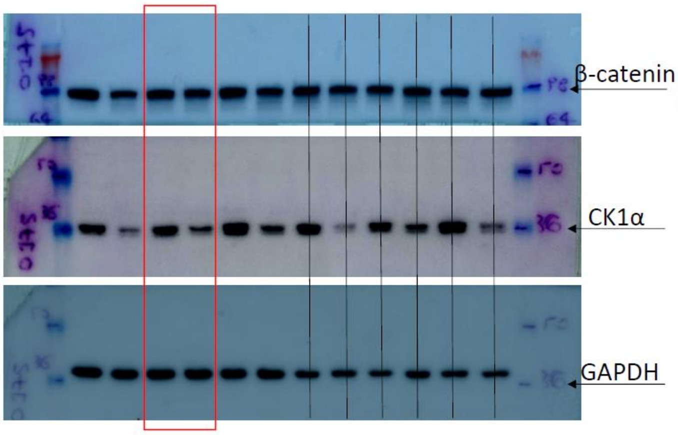

Fig. 1B
6days

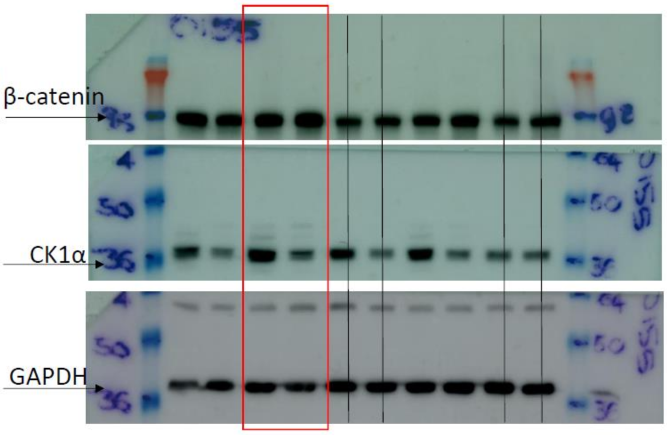

Fig. 1B
11days

Deleted black lanes are related to experiments not presented in the manuscript. Red rectangle indicates the representative WB used for the figure, in an experiment replicated 3 times.

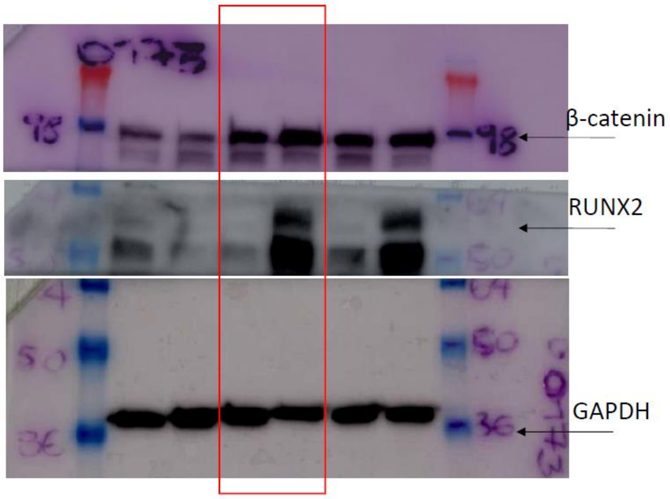

Fig. 2A

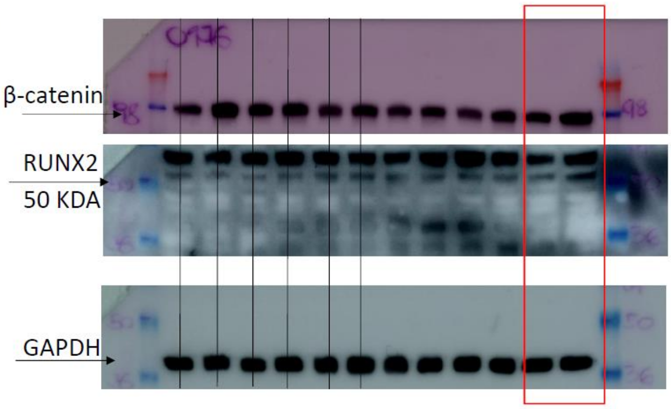

Fig. 2B

Deleted black lanes are related to experiments not presented in the manuscript. Red rectangle indicates the representative WB used for the figure, in an experiment replicated 3 times.

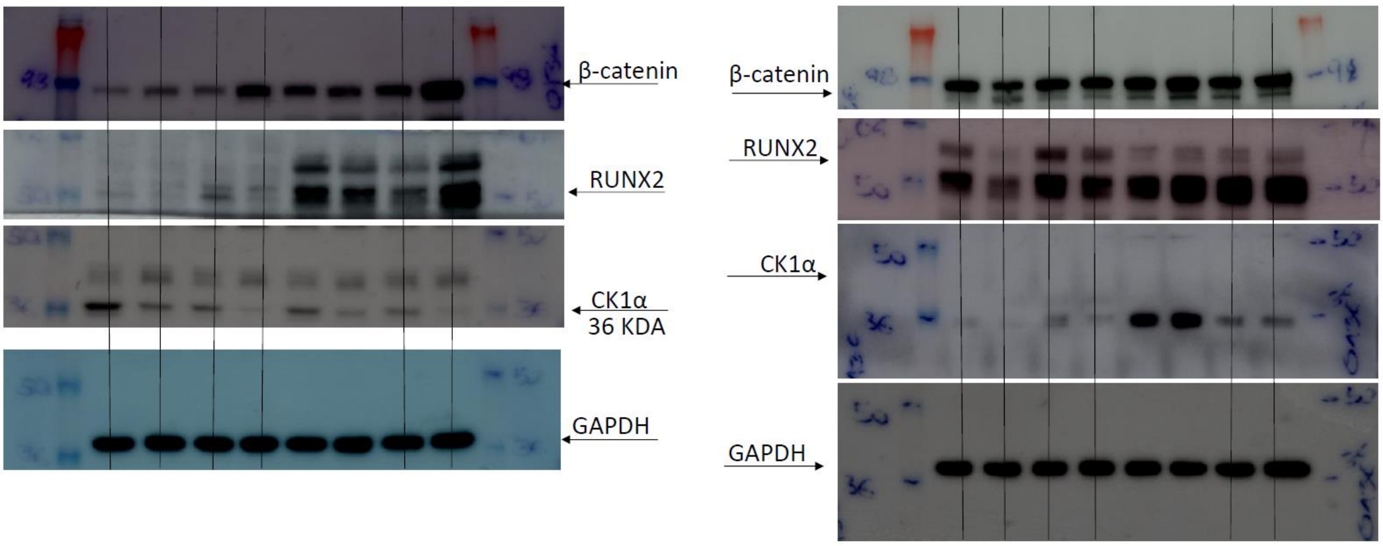

INA-6 6044 **Fig. 3A** **MSC hTERT WT**

Deleted black lanes are related to experiments not presented in the manuscript.

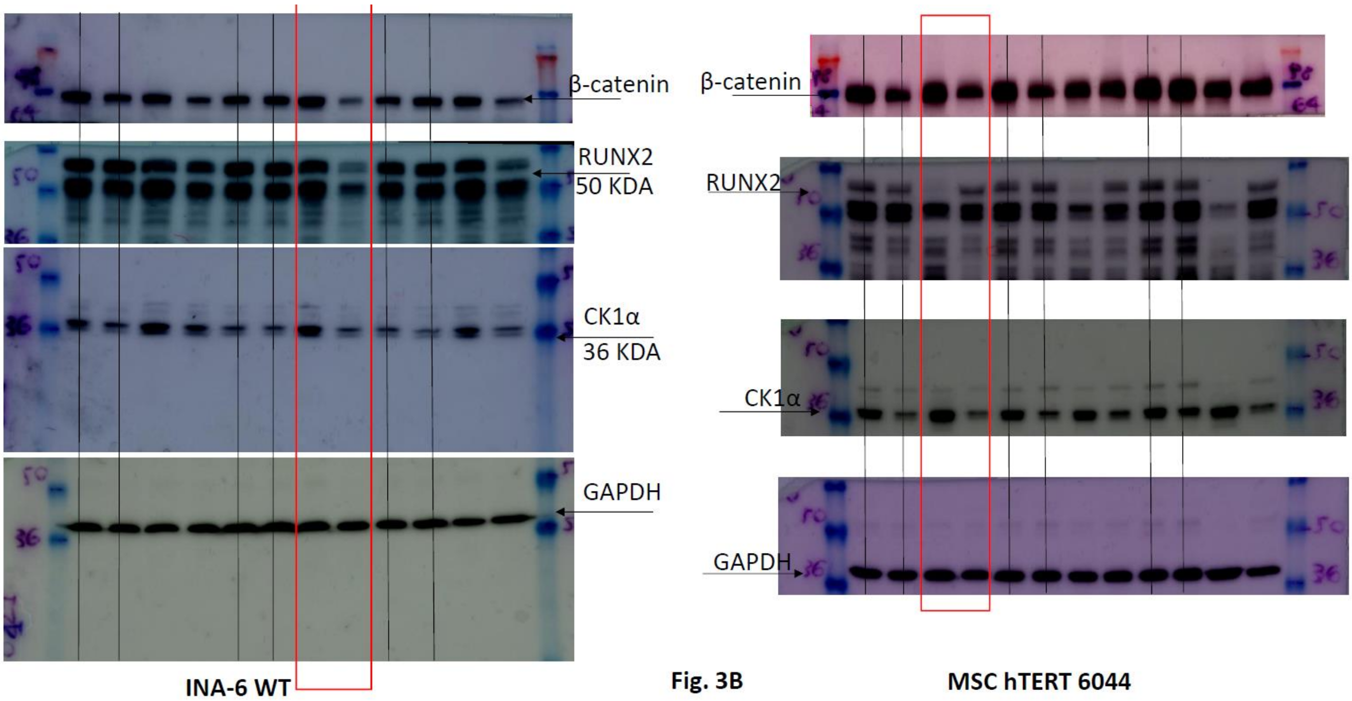

INA-6 WT **Fig. 3B** **MSC hTERT 6044**

Deleted black lanes are related to experiments not presented in the manuscript. Red rectangle indicates the representative WB used for the figure, in an experiment replicated 3 times.

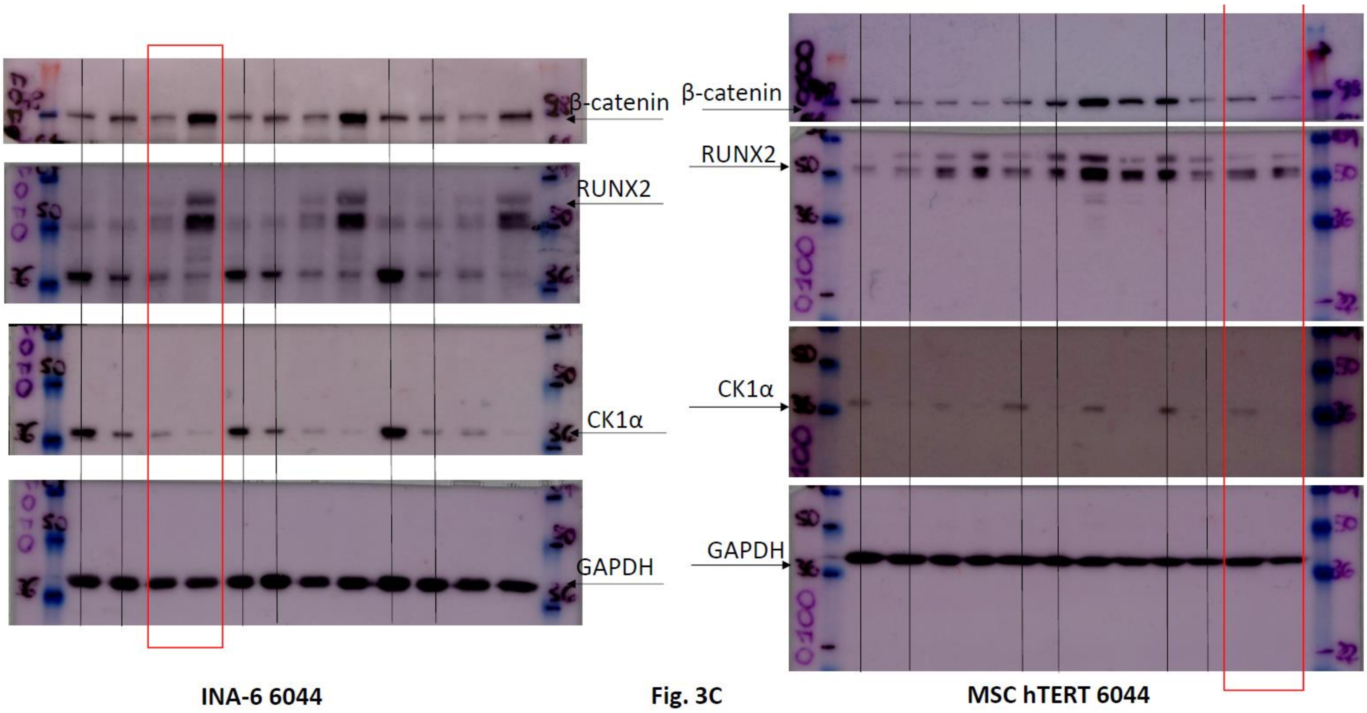

INA-6 6044 **Fig. 3C** **MSC hTERT 6044**

Deleted black lanes are related to experiments not presented in the manuscript. Red rectangle indicates the representative WB used for the figure, in an experiment replicated 3 times.

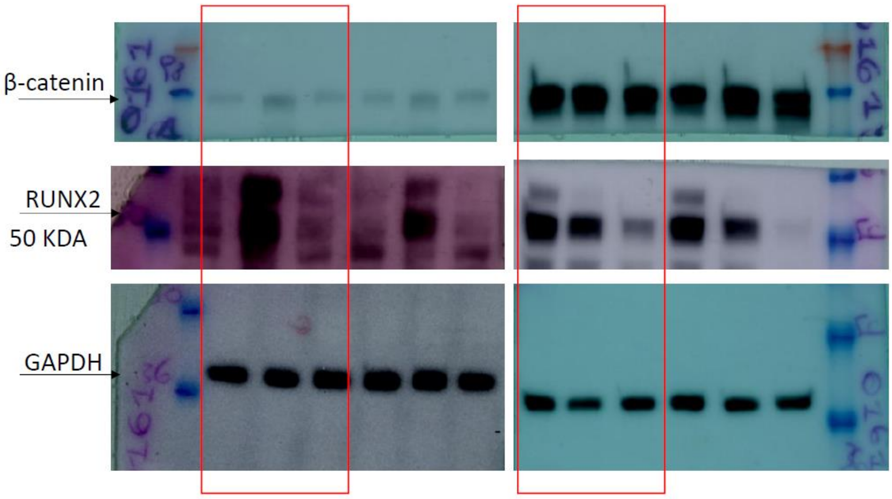

INA-6 6044 **MSC hTERT 6044**

Fig. 4B

Red rectangle indicates the representative WB used for the figure, in an experiment replicated 2 times in this blot.

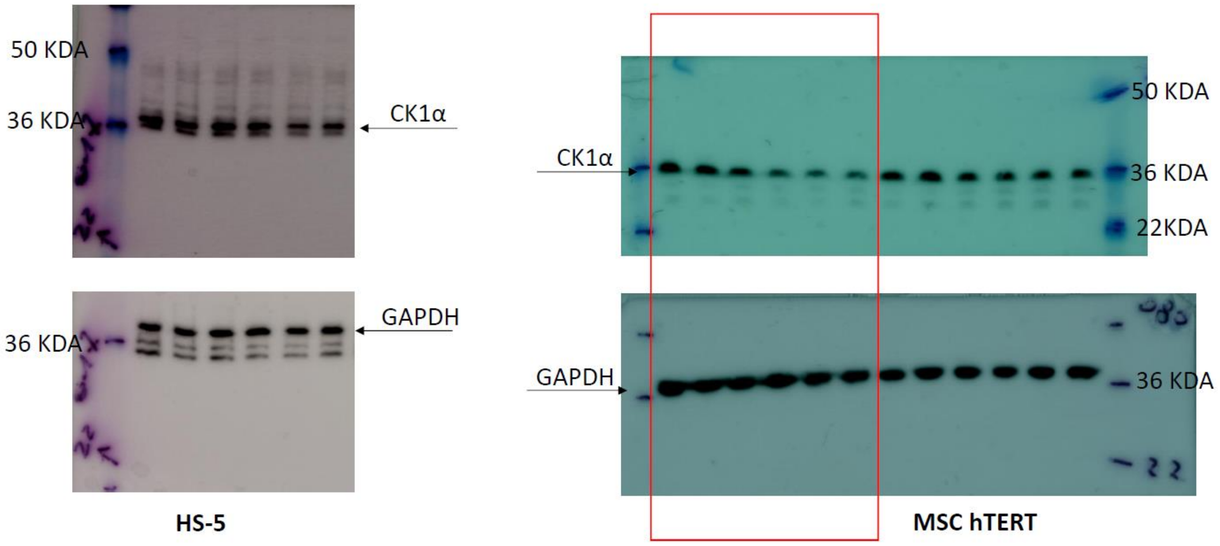

Fig. 5

Red rectangle indicates the representative WB used for the figure in an experiment replicated 2 times in this blot for MSC hTERT cells

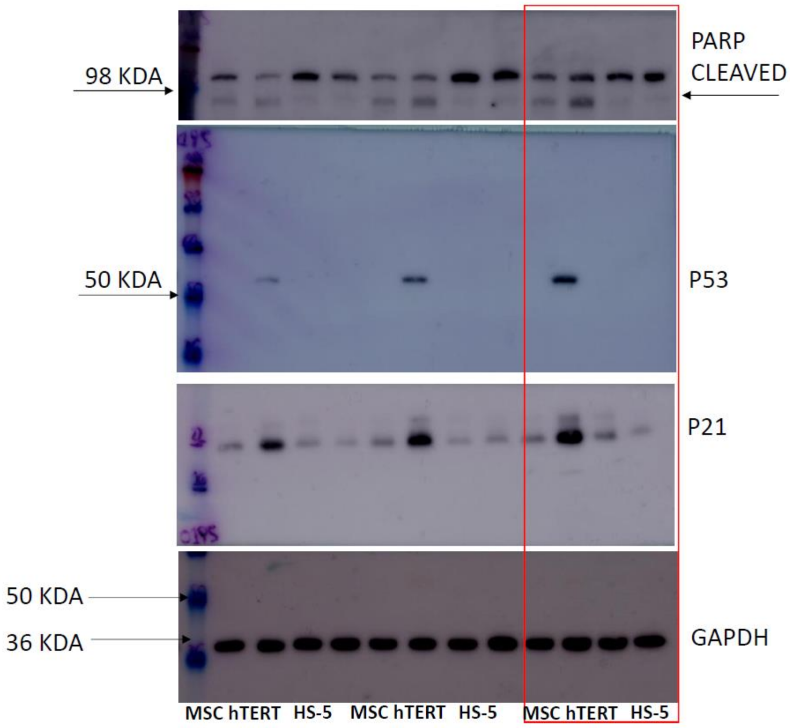

Fig. 6

Red rectangle indicates the representative WB used for the figure, in an experiment replicated 3 times.

Figure S2. Original blots relative to the Western blotting analyses.
